# Osteocytes regulate osteoprotegerin expression via the p38-MAPK-CREB pathway in rheumatoid arthritis

**DOI:** 10.1093/jbmrpl/ziag023

**Published:** 2026-02-17

**Authors:** Keitaro Yasumoto, Toshifumi Fujiwara, Toshiaki Sugita, Shinkichi Arisumi, Shotaro Kawamura, Takeshi Utsunomiya, Daisuke Hara, Shinya Kawahara, Ryosuke Yamaguchi, Yukio Akasaki, Satoshi Hamai, Goro Motomura, Hisakata Yamada, Yasuharu Nakashima

**Affiliations:** Department of Orthopaedic Surgery, Graduate School of Medical Science, Kyushu University, Fukuoka 812-8582, Japan; Department of Orthopaedic Surgery, Graduate School of Medical Science, Kyushu University, Fukuoka 812-8582, Japan; Department of Orthopaedic Surgery, Graduate School of Medical Science, Kyushu University, Fukuoka 812-8582, Japan; Department of Orthopaedic Surgery, Graduate School of Medical Science, Kyushu University, Fukuoka 812-8582, Japan; Department of Orthopaedic Surgery, Graduate School of Medical Science, Kyushu University, Fukuoka 812-8582, Japan; Department of Orthopaedic Surgery, Graduate School of Medical Science, Kyushu University, Fukuoka 812-8582, Japan; Department of Orthopaedic Surgery, Graduate School of Medical Science, Kyushu University, Fukuoka 812-8582, Japan; Department of Orthopaedic Surgery, Graduate School of Medical Science, Kyushu University, Fukuoka 812-8582, Japan; Department of Orthopaedic Surgery, Graduate School of Medical Science, Kyushu University, Fukuoka 812-8582, Japan; Department of Orthopaedic Surgery, Graduate School of Medical Science, Kyushu University, Fukuoka 812-8582, Japan; Department of Orthopaedic Surgery, Graduate School of Medical Science, Kyushu University, Fukuoka 812-8582, Japan; Department of Orthopaedic Surgery, Graduate School of Medical Science, Kyushu University, Fukuoka 812-8582, Japan; Department of Orthopaedic Surgery, Graduate School of Medical Science, Kyushu University, Fukuoka 812-8582, Japan; Department of Orthopaedic Surgery, Graduate School of Medical Science, Kyushu University, Fukuoka 812-8582, Japan

**Keywords:** osteocyte, rheumatoid arthritis, osteoprotegerin, TNF-α, bone remodeling, inflammatory bone loss, p38 MAPK signaling, CREB

## Abstract

Rheumatoid arthritis (RA) is accompanied by systemic and local bone loss, yet the regulation of osteoclast-modulating factors within human osteocytes under inflammatory conditions remains incompletely understood. As an exploratory study, we analyzed surgically resected femoral heads from patients with RA and osteoarthritis (OA). Micro-CT of standardized cancellous regions showed no significant differences in trabecular microarchitectural parameters between groups. In contrast, immunoblot analysis of cortical bone from the femoral neck demonstrated higher osteoprotegerin (OPG) protein levels in RA than in OA. To investigate the underlying mechanism, we examined osteocytes under inflammatory stimulation using the mouse osteocyte-like cell line MLO-Y4 and osteocyte-enriched bone fractions (OEBFs). Among tested cytokines, TNF-α most strongly induced osteoclast-regulatory transcripts and increased OPG protein levels and secretion. TNF-α activated p38-MAPK and cyclic adenosine monophosphate (AMP) response element-binding protein (CREB) signaling, and pharmacologic inhibition of either p38-MAPK activity or CREB activity reduced TNF-α-induced OPG protein accumulation. Chromatin immunoprecipitation (ChIP) further showed enhanced recruitment of CREB to a responsive region within the OPG promoter after TNF-α stimulation. Finally, osteocyte-conditioned medium altered osteoclast morphology and reduced expression of an osteoclast lineage marker during RANKL-driven differentiation, while OPG neutralization did not produce a dominant reversal of these effects. Together, these findings indicate that OPG is upregulated in femoral neck cortical bone from patients with RA and that TNF-α can enhance osteocytic OPG expression via a p38-MAPK-CREB axis, alongside additional osteocyte-derived factors that modulate osteoclast maturation. This study provides evidence from human femoral head specimens of increased OPG in RA cortical bone and suggests that the p38-MAPK-CREB axis may represent an underappreciated regulatory component of osteocyte-derived OPG under inflammatory conditions.

## Graphical Abstract

**Graphical Abstract:**
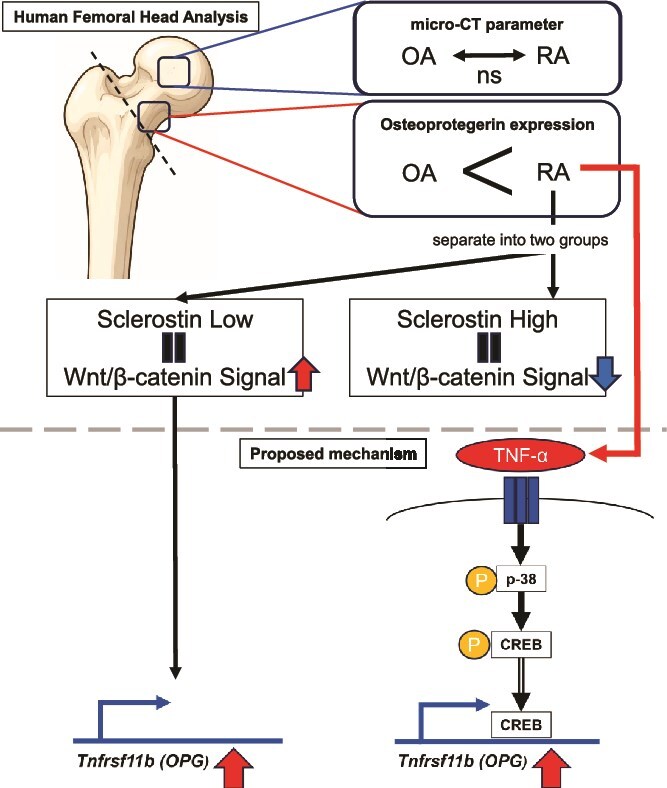

## Introduction

Bone remodeling is maintained by a precisely coordinated cycle of osteoclastic resorption and osteoblastic formation, regulated in large part by the RANK-RANKL-osteoprotegerin (OPG) signaling axis.^1^ Rheumatoid arthritis (RA) is a chronic inflammatory disease in which persistent exposure to pro-inflammatory cytokines induces RANKL and drives osteoclast-mediated local joint destruction, and is frequently accompanied by systemic osteoporosis.^2^ In addition, glucocorticoids—commonly used in RA management—can further compromise skeletal integrity.^3^ As a consequence, patients with RA face an increased risk of fragility fractures, a marked decline in activities of daily living, and substantial healthcare burden.^4–6^ Elucidating how inflammatory signals reshape bone remodeling in RA is therefore a high medical and economic priority.

Osteocytes are uniquely positioned to orchestrate local remodeling because they can express both RANKL and OPG and communicate with osteoblasts and osteoclast precursors through an extensive dendritic network.^7^ Genetic studies have established osteocyte-derived RANKL as a direct trigger of resorption: mice lacking RANKL specifically in osteocytes exhibit markedly suppressed osteoclastogenesis and increased bone mass.^8^^,^^9^ In contrast, evidence from OPG-deficient models under physiological conditions suggests that osteocytic OPG may contribute relatively little to total bone mass,^10^ leaving its functional significance in health and disease unclear. Reduced osteocytic OPG has recently been implicated in the rapid rebound resorption observed after withdrawal of denosumab (anti-RANKL antibody),^11^ a clinical setting distinct from chronic inflammation in RA, suggesting that osteocyte-derived OPG may play a context-dependent role within the local RANK-RANKL-OPG axis. Moreover, pro-inflammatory cytokines, glucocorticoids, ROS, and mechanical unloading have been reported to alter osteocyte function,^7^ further underscoring interest in osteocytic OPG in inflammatory bone loss.

In RA, inflammatory cytokines, such as TNF-α and interleukin (IL)-6, promote RANKL up-regulation in synovial fibroblasts, contributing to bone erosion and joint destruction.^12^ The clinical efficacy of denosumab in RA also highlights the relevance of the RANK-RANKL-OPG axis.^1^ Nevertheless, the impact of inflammatory cytokines on osteocytic OPG has barely been examined. Pathak et al.^13^ reported that RA serum or individual IL-1β/TNF-α did not change OPG mRNA in human trabecular bone explants, whereas Marahleh et al.^14^ observed increased RANKL but unchanged OPG in mouse osteocytes exposed to TNF-α. These findings are consistent with the concept that osteoblast-derived OPG is a major protective factor in trabecular bone,^10^ yet they leave unresolved whether osteocytic OPG is differentially regulated in cortical bone and/or under RA-associated inflammatory stress in vivo. Collectively, these observations motivated us to investigate osteocyte-derived OPG regulation under inflammatory conditions using complementary human and experimental models.

To fill this knowledge gap, we first conducted an exploratory analysis of osteocyte-related regulators (RANKL, OPG, and sclerostin) in cortical bone specimens obtained from femoral heads of patients with RA and osteoarthritis (OA). Studies directly interrogating osteocyte-derived regulators of the RANK-RANKL-OPG axis in human bone remain scarce, and the acquisition of truly healthy human bone is ethically and practically challenging. Thus, while recognizing that OA does not represent a completely non-inflammatory control, comparing RA with OA provides a pragmatic clinical framework to contextualize RA-associated osteocyte-related molecular changes beyond macroscopic bone architecture. In this exploratory human analysis, we observed that osteocytic OPG expression tended to be elevated in RA. Because observations from clinical specimens may reflect not only inflammation but also concomitant medications—including disease-modifying therapies and anti-osteoporosis therapies—we next established experimental systems using a mouse osteocyte-like cell line and osteocyte-enriched bone fractions (OEBFs) from long bones to examine the direct effects of inflammatory cytokines. In these models, TNF-α induced OPG (along with RANKL), and signaling analyses suggested activation of the inflammation-responsive p38-MAPK-cAMP response element-binding protein (CREB) pathway in addition to canonical Wnt/β-catenin signaling upstream of OPG regulation. Together, these data suggest that inflammatory cues can induce an osteocytic OPG response via p38-MAPK-CREB signaling. This response may represent a compensatory mechanism under inflammatory stress; however, whether it is sufficient to counterbalance concomitant pro-resorptive signals and how it influences net bone remodeling in vivo warrant further investigation.

## Materials and methods

### Human specimens

This study was conducted in accordance with the principles of the Declaration of Helsinki. The study protocol was reviewed and approved by the Regional Committee of Ethics for Human Research at the Faculty of Medicine of Kyushu University (approval number: 22314-00). Written informed consent was obtained from all participants prior to enrollment, and all participants provided signed consent forms indicating voluntary participation.

A total of 39 Japanese patients who underwent total hip arthroplasty at Kyushu University Hospital between September 2020 and July 2025 were enrolled, including 25 with RA and 14 with OA. All RA patients fulfilled the 1987 American College of Rheumatology (ACR) classification^15^ or the 2010 ACR/European Alliance of Associations for Rheumatology (EULAR) classification criteria.^16^ Resected femoral heads were promptly subjected to micro-CT. The periosteum and trabecular bone of the femoral neck were removed using a rongeur, and cortical bone was collected piece by piece and stored at −80 °C until analysis. Clinical information, such as demographic data (age, sex, height, weight, and disease duration), laboratory values (rheumatoid factor [RF], anti-citrullinated protein/peptide antibody, and serum C-reactive protein [CRP]), and medications for RA and osteoporosis were obtained from medical records.

### Micro-CT

**Figure 1:**
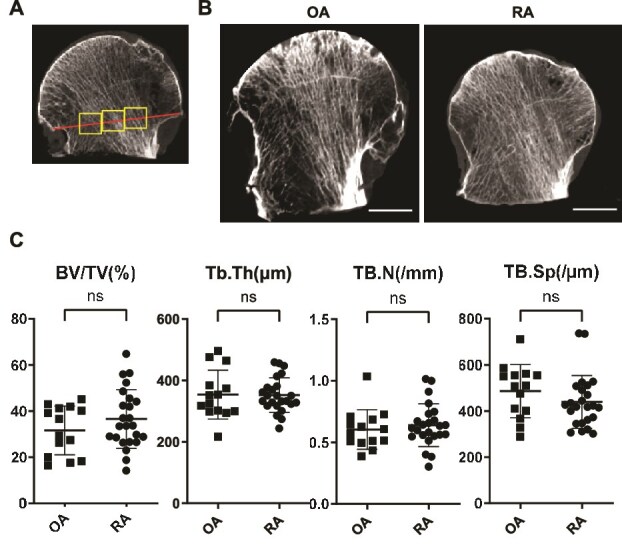
Micro-CT analysis of femoral-head bone microarchitecture demonstrates comparable trabecular structural properties between patients with rheumatoid arthritis (RA) and osteoarthritis (OA). (A) Schematic illustration of the ROI used for micro-CT analysis of the femoral head. ROIs were placed in 3 standardized locations along a line connecting the inflection points of the femoral head contour in the central sagittal slice. Regions affected by articular surface collapse or surgical puncture were excluded, and ROI placement was adjusted to account for inter-individual variability in femoral neck resection length. (B) Representative sagittal micro-CT images of femoral heads from patients with RA and OA, illustrating bone microarchitecture in sections including the analyzed ROI. Scale bars are 10 mm. (C) Dot plots of trabecular microarchitecture parameters, including bone volume fraction (BV/TV), trabecular thickness (Tb.Th), trabecular number (Tb.N), and trabecular separation (Tb.Sp), in RA (*n* = 25) and OA (*n* = 14) specimens. Data are shown as individual values with mean ± SD; ns indicates not significant by 2-sided Student’s *t*-test.

All resected femoral heads were promptly scanned using high-resolution micro-CT (R_mCT T, Rigaku). The scanning parameters were set at a voltage of 60 kV, current of 60 μA, resolution of 50 μm per pixel, and slice thickness of 0.4 mm. ROI were placed in 3 areas per specimen along a line connecting the inflection points of the femoral head contour in the central sagittal slice, avoiding sites of collapse or surgical puncture and accounting for variations in femoral neck resection length (Figure 1A). Each ROI was defined as a cube measuring 5 × 5 × 5 mm (38 × 38 × 38 voxels). Three-dimensional structural parameters were assessed using a 3D image analysis system (TRI/3D-BON, RATOC System Engineering) and a calibration phantom (Kyoto Kagaku). The following trabecular parameters were quantified and compared between the RA and OA groups: bone volume fraction (BV/TV), trabecular thickness (Tb.Th), trabecular number (Tb.N), and trabecular separation (Tb.Sp).^17^

### Mouse models and preparation of OEBFs

All animal procedures were reviewed and approved by the IACUC at Kyushu University (approval number: A23-004-0) and were conducted in strict accordance with the National Institutes of Health guidelines for the ethical treatment and use of laboratory animals.

Osteocyte-enriched bone fractions were prepared as previously described by Yan et al.,^18^ with minor modifications. Briefly, femurs and tibiae were harvested from 8- to 10-wk-old C57BL/6J mice, in which both male and female animals were included and no sex-based stratification was performed. After removing both proximal and distal ends, the bone marrow was flushed out by centrifugation and stored for downstream experiments. The outer bone surface was scraped with a scalpel, and the bones were roughly fragmented into small pieces. Bone fragments were digested in α-MEM containing 0.1% collagenase (038-22 361, FUJIFILM Wako) and 0.2% Dispase II (383-02281, FUJIFILM Wako) at 37 °C with shaking for 6 consecutive 15-min cycles. After digestion, osteocyte-enriched tissues were cultured in α-MEM supplemented with 10% FBS, 100 IU/mL penicillin, and 100 μg/mL streptomycin. Following serum starvation, the cells were stimulated with TNF-α (100 ng/mL, R&D Systems) for 24-48 h and then harvested for subsequent qRT-PCR and immunoblot analyses.

### Cell culture

The mouse osteocyte cell line MLO-Y4 was kindly provided by Dr. Kosaku Kurata (Kyushu University) with permission from Prof. Lynda Bonewald (Indiana University School of Medicine). Cells were cultured in α-minimum essential medium supplemented with 2.5% FBS, 2.5% iron-supplemented calf serum, 100 IU/mL penicillin, and 100 μg/mL streptomycin, and maintained at 37 °C in a humidified incubator with 5% CO₂. For gene expression analysis, MLO-Y4 cells were seeded at a density of 6 × 10^4^ cells per well in 6-well plates and cultured to 70%-80% confluence. After serum starvation, the cells were treated with TNF-α (10 ng/mL) for 24 h and then harvested. For protein expression analysis, cells were seeded at a density of 1 × 10^5^ cells per 10-cm dish, cultured to 70%-80% confluence, subjected to the same serum starvation protocol, and treated with TNF-α (10 ng/mL) for 24 h prior to collection. For inhibitor experiments, the p38 MAPK inhibitor SB203580 (Selleck Chemicals) was used at a concentration of 30 μM, and the CREB inhibitor 666-15 (Selleck Chemicals) was used at a concentration of 0.1 μM. Inhibitors were added according to the experimental design in the presence of TNF-α stimulation.

### RNA isolation and quantitative real-time PCR

Total RNA was extracted from MLO-Y4 cells and osteoclasts using the RNeasy Mini Kit (Qiagen) according to the manufacturer’s instructions. RNA extraction from OEBFs was performed following the method described by Kelly et al.^19^ Briefly, OEBFs were pulverized in liquid nitrogen using a CryoMill, homogenized in TRIzol reagent (Thermo Fisher Scientific), and incubated at room temperature for 45 min. Phase separation was achieved by adding 300 μL chloroform, followed by vortexing and centrifugation at 12 000 × *g* for 10 min at 4 °C. The aqueous phase was mixed with an equal volume of 70% ethanol and further purified using the RNeasy Mini Kit (Qiagen). cDNA was synthesized from 0.1 μg of total RNA using the PrimeScript RT reagent kit (Takara Bio). Quantitative PCR was performed using TaqMan Gene Expression Assays (Thermo Fisher Scientific) for the following targets: *Tnfsf11* (Mm00441906_m1), *Tnfrsf11b* (Mm00435454_m1), *Sost* (Mm00470479_m1), *Ctnnb1* (Mm00483039_m1), *Axin2* (Mm00443610_m1), *Lef1* (Mm00550265_m1), and *Acp5* (Mm00475-698_m1). *Gapdh* (Mm99999915_g1) was used as the endogenous control for MLO-Y4 cells and OEBFs, whereas *Mrps2* (Mm00475529_m1) was used for normalization in osteoclast samples.^20^ Gene expression was normalized using the ΔΔCt method, with unstimulated samples used as the calibrator. Ct values were carefully examined, and signals with Ct values above 35 were considered near the detection limit and excluded from quantitative analyses. All reactions were performed in triplicate.

### Protein extraction and immunoblotting

Proteins were extracted from human femoral heads (0.1 g) and OEBFs (0.04 g) after washing with PBS and pulverizing in liquid nitrogen using a CryoMill. Bone powder was lysed in Radioimmunoprecipitation assay (RIPA) buffer (Thermo Scientific) supplemented with protease inhibitor cocktail (cOmplete Mini, Sigma-Aldrich), incubated on ice for 30 min, and heat-denatured at 99 °C for 10 min using a thermal cycler. Lysates were centrifuged at 14 000 × *g* for 15 min at 4 °C, and the supernatant was collected for analysis. For MLO-Y4 cells, protein extraction was performed by washing the cells with PBS, lysing them in the same RIPA buffer with protease inhibitors, incubating them on ice for 30 min, and centrifuging at 14 000 × *g* for 15 min at 4 °C. The resulting supernatants were used for the downstream experiments. Protein concentrations were determined using a Pierce Dilution-Free Rapid Gold BCA Protein Assay Kit (Thermo Fisher Scientific) and an iMark Microplate Reader (Bio-Rad). Equal amounts of protein (10-30 μg) were separated on 4%-12% gradient SDS-PAGE gels (Invitrogen) and transferred onto polyvinylidene difluoride membranes (Amersham Biosciences) using a semi-dry blotting system (Bio-Rad). Membranes were blocked with 5% non-fat dry milk in Tris-buffered saline for 1 h and incubated overnight at 4 °C with primary antibodies (listed below). Horseradish peroxidase-conjugated secondary antibodies (Santa Cruz Biotechnology) were then applied. Immunoreactive bands were detected using ECL Prime (Amersham Biosciences), following three washes with TBS containing 0.1% Tween 20, and images were captured using an Ez Capture MG system (ATTO). Band intensities were quantified using CS Analyzer 3.0 software (ATTO).^21^

The following primary antibodies were purchased: anti-CREB (#4820), anti-phospho-CREB (#9198), anti-p38 MAPK (#9212), anti-phospho-p38 MAPK (#9211), anti-JNK (#9252), anti-phospho-JNK (#9251), anti-ERK1/2 (#9102), and anti-phospho-ERK1/2 (#9106) were purchased from Cell Signaling Technology; anti-osteoprotegerin (ab9986) and anti-sclerostin (ab63097) were from Abcam; and anti-β-actin (#A00702) was from GenScript.

### ELISA

Osteoprotegerin concentrations in MLO-Y4 culture supernatants were quantified 24 h after TNF-α stimulation using a sandwich Mouse Osteoprotegerin/TNFRSF11B ELISA Kit (MOP00, R&D Systems) according to the manufacturer’s instructions. The supernatants were cleared by centrifugation and diluted 1:2 in the kit sample diluent. A standard curve was generated with the lyophilized standards supplied in the kit, and the absorbance was recorded at 450 nm with 620 nm reference correction using an iMark microplate reader (Bio-Rad). Osteoprotegerin concentrations were calculated using a 4-parameter logistic (4 PL) fit of the standard curve. All samples were assayed in duplicate.

### Chromatin immunoprecipitation assay

Chromatin immunoprecipitation (ChIP) experiments were performed using the SimpleChIP Enzymatic Chromatin IP Kit (#9002; Cell Signaling Technology), according to the manufacturer’s instructions. MLO-Y4 cells were serum-starved for 1 h and then stimulated with TNF-α (10 ng/mL) for 24 h. Protein-DNA complexes were cross-linked with formaldehyde, nuclei were isolated, and chromatin was enzymatically digested to the desired fragment size. An aliquot of each sample was used as the input control, and the remaining chromatin was incubated for 24 h with an anti-CREB antibody (#4820, Cell Signaling Technology). Immunoprecipitated complexes were treated with proteinase K, and the recovered DNA was amplified by PCR using primer pairs designed to generate ~100-bp products spanning the 2-kb promoter region of the *Opg* gene.

### Osteoclast differentiation and TRAP staining

Bone marrow-derived macrophages (BMMs) were depleted of erythrocytes by incubation for 5 min at room temperature in lysis buffer containing 150 mM NH₄Cl, 10 mM KHCO₃, and 0.1 mM EDTA (pH 7.4). After washing, 5 × 10^6^ BM cells were plated onto 100-mm dishes and cultured for 4-5 d in α-MEM supplemented with 10% heat-inactivated FBS and 1% penicillin-streptomycin containing 10% CMG 14-12 conditioned medium (CM), which provided ~1 μg/mL recombinant M-CSF.^22^ The medium and CMG 14-12 supernatant were replaced every other day. On culture day 3, BMMs were replated in 96-well or 6-well plates and induced to form osteoclasts by adding 1% CMG 14-12 supernatant together with 100 ng/mL recombinant RANKL (Oriental Yeast).

To generate osteocyte-conditioned media, MLO-Y4 cells were stimulated with TNF-α (10 ng/mL) for 24 h. The TNF-α-containing medium was then removed, cells were washed twice with PBS, and fresh TNF-α-free α-MEM (10% FBS, 1% penicillin-streptomycin) was added. After an additional 24 h, the supernatant was collected as TNF-α-stimulated osteocyte-CM (TNF-stim CM). This PBS-wash and medium-replacement step was performed to remove TNF-α and minimize direct carry-over of TNF-α into subsequent osteoclast cultures. In parallel, CM from unstimulated MLO-Y4 cells (Unstim CM) was prepared identically without TNF-α exposure. For osteoclast differentiation assays, the following 5 conditions were compared (all containing 100 ng/mL RANKL): (1) no MLO-Y4 CM and no TNF-α (RANKL only; no TNF source); (2) TNF-α added directly to osteoclast cultures (TNF-α, no MLO-Y4); (3) Unstim CM added at 10% (v/v); (4) TNF-stim CM added at 10% (v/v); and (5) TNF-stim CM pre-incubated with a neutralizing anti-OPG antibody (AF459, R&D Systems) prior to addition to osteoclast cultures. After 4-5 d, osteoclast formation was confirmed. Cells were either harvested for RNA extraction and subsequent gene expression analysis or fixed with 4% paraformaldehyde in PBS. Tartrate-resistant acid phosphatase (TRAP) staining was performed as described previously, using sodium-potassium tartrate and Naphthol AS-BI phosphate (Sigma Aldrich).^23^

### Statistics

Data are presented as mean ± SD. Normality was assessed using the Shapiro–Wilk test. For 2-group comparisons, normally distributed data were analyzed with a 2-tailed Student’s *t*-test, and non-normal data were analyzed using Mann–Whitney U test. Categorical variables were compared using the chi-squared test. Comparisons among 3 or more groups were performed using one-way ANOVA followed by Tukey’s post hoc test. All analyses were conducted using Prism version 10.6.1 (GraphPad Software). Statistical significance was defined as a 2-sided *p*-value <.05.

## Results

### OPG expression was upregulated in femoral neck cortex from patients with RA

The clinical characteristics of the collected femoral neck cortex are summarized in Table 1. No significant differences were detected between the two groups in any comparable parameters.

**Table 1:** Baseline characteristics of RA and OA femoral-head donors.

Variable	RA	OA	*p*
*N*	25	14	–
Age (yr), median (range)	68 (47–84)	69 (48-85)	.98
Female, *N* (%)	22 (88.0)	14 (100)	.54
BMI, median (range)	24.2 (17.1–30.1)	22.5 (19.0–26.9)	.18
Disease duration (yr), median (range)	11 (1–46)	–	–
RF (IU/mL), median (range)	17.5 (0–220)	–	–
CRP (mg/L), median (range)	0.1 (0.01–8.26)	0.06(0.01–1.38)	.10
Steinbrocker stage, *N* (%)	IV: 4 (18) III: 6 (27) II: 10 (45) I: 2 (9)	–	–
Treatment			
MTX use, *N* (%) MTX dose (mg), median (range)	14 (56) 7 (2–16)	– –	– –
GC use, *N* (%) GC dose (mg), median (range) b/ts DMARDs use, *N* (%) TNF-α inhibitor, *N* (%) IL-6 inhibitor, *N* (%) CTLA4Ig, *N* (%) JAK inhibitor, *N* (%)	10 (38) 5 (1–13) 12 (48) 7 (58) 1 (82) 1 (8) 2 (17)	1 (14) 1 (6) –	– –
Anti-osteoporosis drug use, *N* (%)	11 (44)	2 (14)	–
Vitamin D, *N* (%)	3 (27)	1 (50)	–
SERM, *N* (%)	1 (9)	–	–
Bisphosphonate, *N* (%)	4 (36)	1 (50)	–
Denosumab, *N* (%)	2 (18)	–	–
rPTH, *N* (%)	1 (9)	–	–
Romosozumab, *N* (%)	1 (9)	–	–

Abbreviations: b/ts DMARDs, bio/targeted synthetic disease-modifying anti-rheumatic drugs; CRP, C-reactive protein; GC, glucocorticoid; IL-6, interleukin-6, JAK, janus kinase; MTX, methotrexate; RF, rheumatoid factor; rPTH, recombinant PTH.

Next, we performed micro-CT to analyze trabecular bone microarchitecture in the femoral head using predefined ROI, as schematically illustrated in Figure 1A. Briefly, ROIs were placed in three standardized locations—central, medial, and lateral—along a line connecting the inflection points of the femoral head contour in the central sagittal slice. This ROI placement strategy was designed to minimize variability arising from differences in femoral neck resection planes among specimens and to avoid regions affected by surgical extraction puncture. Representative micro-CT images of the analyzed regions are shown in Figure 1B. Quantitative analysis demonstrated no significant differences between RA and OA samples in bone mass or trabecular structural parameters, including BV/TV, Tb.Th, Tb.N, and Tb.Sp (Figure 1C). Specifically, BV/TV, Tb.Th, Tb.N, and Tb.Sp were comparable between the RA and OA groups, with no parameter reaching statistical significance.

**Figure 2:**
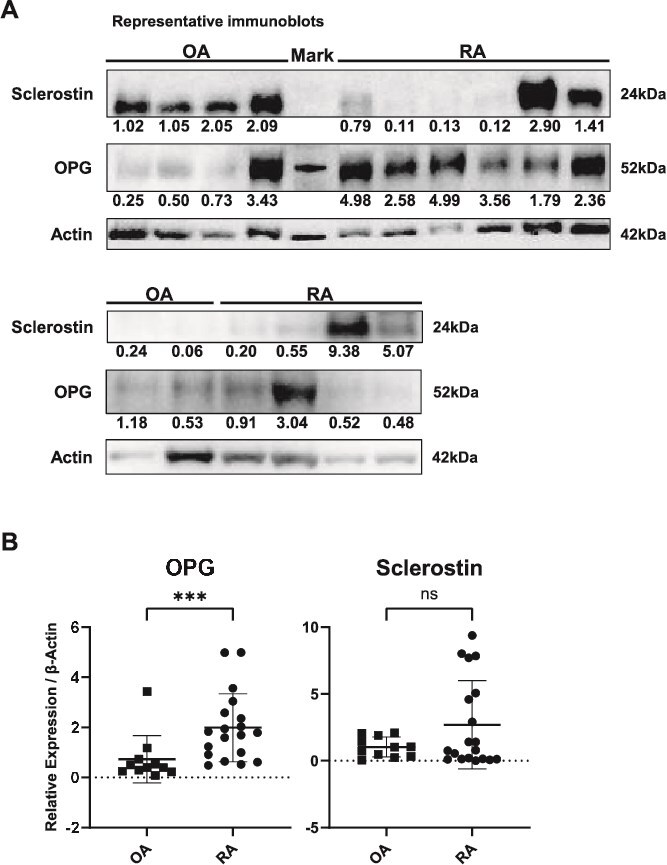
Osteoprotegerin expression is increased in femoral heads from patients with RA compared with OA. (A) Representative immunoblot of OPG and sclerostin from a subset of RA and OA specimens. Densitometric quantification was performed using all available samples (RA: *n* = 19, OA: *n* = 11); β-actin serves as the loading control. Numeric values shown beneath each band represent the relative expression levels of sclerostin/β-actin or OPG/β-actin for the corresponding lane. (B) Dot plots of densitometric quantification of OPG (normalized to β-actin) in RA (*n* = 19) and OA (*n* = 11) specimens. Data are shown as individual values with mean ± SD; ^***^p < .001; ns indicates not significant by the 2-sided Mann–Whitney U test.

Protein analyses demonstrated a significant increase in OPG expression in cortical bone from patients with RA compared with OA, whereas sclerostin levels were comparable between the 2 groups (Figure 2A and B).

Next, we conducted statistical analyses to determine whether the treatments patients received for RA or osteoporosis were associated with parameters obtained from micro-CT and western blotting. In univariable analyses with biologic and targeted synthetic disease-modifying antirheumatic drugs (b/ts DMARDs) use as the explanatory variable, significant associations were observed for disease duration, RF, and CRP (Table S1). Patients receiving b/ts DMARDs had significantly longer disease duration, higher preoperative RF levels, and lower preoperative CRP values. In contrast, in the analysis using antiresorptive drugs (bisphosphonates, SERMs, or denosumab) as the explanatory variable, no significant associations were identified for any parameter (Table S2).

### TNF-α causes the greatest change in bone metabolism-related genes in bone cells

**Figure 3:**
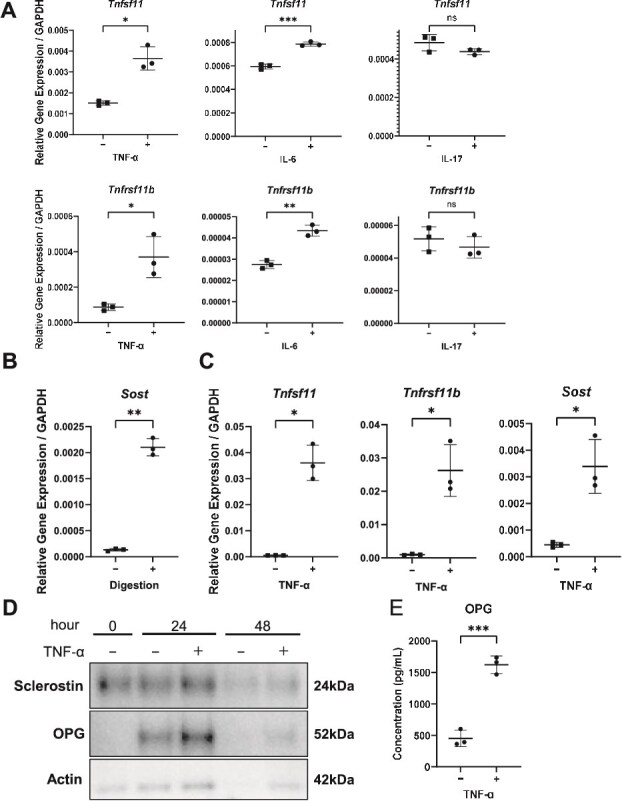
TNF-α most strongly modulates osteocyte-related gene and protein expression. (A) Dot plots of the relative mRNA levels for *Tnfsf11* (RANKL) and *Tnfrsf11b* (OPG) in MLO-Y4 cells stimulated with TNF-α, IL-6 or IL-17 (*n* = 3). (B) Dot plots showing enrichment of the osteocyte marker *Sost* in osteocyte-enriched bone fraction (OEBFs) vs non-digested bone (*n* = 3). (C) Dot plots of *Tnfsf11*, *Tnfrsf11b*, and *Sost* mRNA in OEBF with or without TNF-α (*n* = 3). (D) Representative immunoblot of OPG and Sclerostin in OEBF with or without TNF-α; β-actin serves as the loading control. (E) Dot plots of secreted OPG in conditioned medium from MLO-Y4 cells treated with TNF-α, measured by ELISA (*n* = 3). Data in (A-C and E) are individual values with the mean ± SD; ^*^p < .05, ^**^p < .01, ^***^p < .001; ns indicates not significant by the 2-sided Student’s *t*-test.

To determine which major RA-associated pro-inflammatory cytokines exert the greatest influence on osteocytes, the mouse osteocytic cell line MLO-Y4 was stimulated with TNF-α, IL-6, or IL-17, and changes in bone metabolism-related gene expression were analyzed. Among the cytokines tested, TNF-α induced the most pronounced up-regulation of both RANKL and OPG mRNA, whereas IL-6 exerted more modest effects and IL-17 produced no significant changes (Figure 3A).

Because MLO-Y4 cells express low basal levels of Sost and are therefore suboptimal for evaluating its regulation, we next employed an ex vivo culture system using OEBFs. Enzymatic enrichment markedly increased Sost expression, confirming successful osteocyte enrichment (Figure 3B). In OEBFs, TNF-α stimulation robustly up-regulated RANKL, OPG, and Sost expression at the mRNA level (Figure 3C). Protein analyses corroborated these transcriptional findings, demonstrating increased OPG protein levels in OEBFs after TNF-α stimulation (Figure 3D). Consistently, ELISA analysis revealed a marked increase in OPG secretion from TNF-α-stimulated MLO-Y4 cells (Figure 3E), whereas soluble RANKL in the CM remained below the detection limit under these experimental conditions (data not shown).

Collectively, these results indicate that TNF-α most robustly induces the expression of osteoclast-regulatory factors in osteocytes and is likely a principal driver of inflammatory dysregulation of bone metabolism in RA.

### TNF-α activates p38-MAPK-CREB signaling and enhances CREB recruitment to the OPG promoter

**Figure 4:**
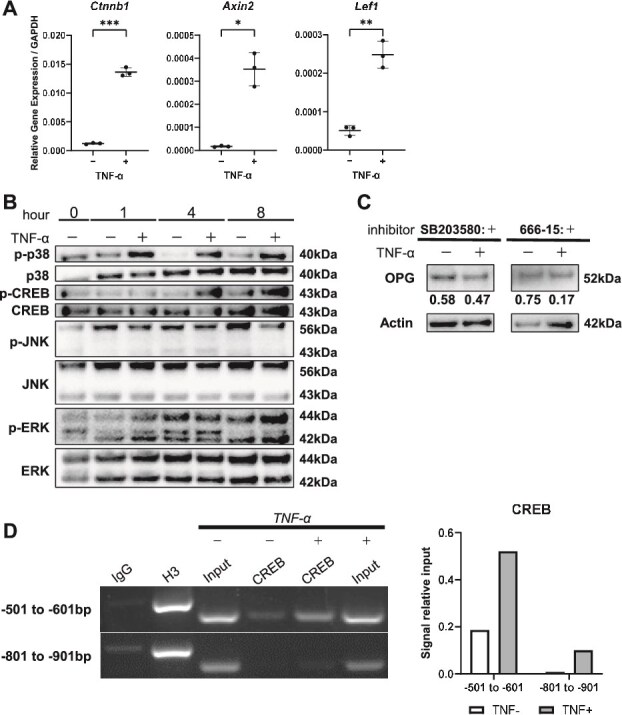
TNF-α-induced changes in Wnt/β-catenin-related transcripts and activation of the p38-MAPK-CREB signaling pathway in osteocytes. (A) Dot plots showing relative mRNA levels of *Ctnnb1* (β-catenin), *Lef1*, and *Axin2* in MLO-Y4 cells treated with or without TNF-α (*n* = 3). (B) Representative immunoblotting images showing phosphorylated p38 MAPK (p-p38), total p38, phosphorylated CREB (p-CREB), total CREB, phosphorylated JNK (p-JNK), total JNK, phosphorylated ERK (p-ERK), total ERK, and β-actin in MLO-Y4 cells following TNF-α treatment. Data in (A) are shown as individual values with the mean ± SD and were analyzed by the two-sided Student’s *t*-test. (C) Representative immunoblot of OPG in TNF-α-stimulated MLO-Y4 cells treated with a p38 inhibitor or a CREB inhibitor. Cells were stimulated with TNF-α in the presence or absence of SB203580 (p38 inhibitor) or 666-15 (CREB inhibitor), and total cell lysates were subjected to immunoblotting for OPG. β-actin serves as the loading control. Numeric values shown beneath each band represent the relative expression levels of OPG/β-actin for the corresponding lane. (D) ChIP-PCR demonstrating enrichment of the OPG promoter in anti-CREB immunoprecipitates from MLO-Y4 cells with or without TNF-α stimulation, with densitometric quantification of the ChIP-PCR bands; input DNA served as the internal control, normal IgG as the negative control, and anti-histone H3 as the positive control.

To elucidate the mechanisms governing OPG expression in osteocytes, we first examined the sclerostin-associated Wnt/β-catenin axis in OEBFs. At a single time point, TNF-α increased the expression of Wnt/β-catenin-related transcripts, including *Ctnnb1*, *Axin2*, and *Lef1* (Figure 4A). As shown in the preceding section, TNF-α stimulation also increased Sost expression in OEBFs at a single time point. Because sclerostin is a potent inhibitor of Wnt/β-catenin signaling, we next evaluated the time-dependent relationship between Sost and Wnt target genes under TNF-α stimulation. In OEBFs, *Sost* expression was transiently high at early time points and then declined markedly, whereas *Lef1* and *Axin2* exhibited time-dependent changes with a late-phase increase, most prominently for *Axin2* at 48 h (Figure S1). Thus, in this ex vivo system, *Sost* and Wnt target gene expression did not change in parallel, underscoring time- and context-dependent dynamics of these transcripts in response to TNF-α.

By contrast, in our human femoral neck cortical bone data, high OPG expression was observed even in some samples with high sclerostin levels, suggesting that osteocytic OPG regulation is not solely explained by the sclerostin/Wnt axis. Previous studies have shown that, in bone marrow stromal cells (BMSCs), OPG expression is regulated by the inflammation-responsive p38-MAPK-CREB cascade.^24^ We therefore hypothesized that a similar mechanism operates in terminally differentiated osteocytes and evaluated the behavior of this signaling pathway in response to TNF-α stimulation. Consistently, TNF-α induced phosphorylation of p38 and CREB in MLO-Y4 cells (Figure 4B), supporting activation of the p38-MAPK-CREB pathway. Collectively, these results suggest that inflammatory signaling via p38-MAPK-CREB contributes to osteocytic OPG regulation, alongside potential contributions from homeostatic pathways.

To functionally assess whether p38 and CREB activity contributes to TNF-α-induced OPG expression in osteocytes, we performed pharmacological inhibition experiments under TNF-α stimulation in MLO-Y4 cells. Treatment with the p38 inhibitor SB203580 or the CREB inhibitor 666-15 reduced OPG protein levels compared with the corresponding TNF-α-treated vehicle controls (Figure 4C). These observations provide functional support that the TNF-α-responsive p38-CREB axis contributes to osteocytic OPG regulation.

To assess whether TNF-α enhances CREB recruitment to the OPG promoter in osteocytes, we conducted ChIP assays. In the previously identified CREB-responsive region within the OPG promoter (−501 to −601 bp),^24^ CREB occupancy was significantly higher than that in a distinct region within the same OPG promoter (−801 to −901 bp) and was further increased by TNF-α stimulation (Figure 4D).

Collectively, these results support a model in which TNF-α-responsive p38-MAPK-CREB signaling contributes to osteocytic OPG regulation, in a context where sclerostin/Wnt-β-catenin signaling alone does not fully account for OPG expression patterns.

### Osteocyte-CM modulates osteoclast morphology and Acp5 expression under RANKL stimulation

To assess the effects of osteocyte-derived soluble factors on osteoclastogenesis, BMMs were differentiated into osteoclasts in the presence of recombinant RANKL and cultured under 5 conditions: RANKL alone, direct TNF-α treatment, conditioned medium (CM) from unstimulated MLO-Y4 cells, CM from TNF-α-stimulated MLO-Y4 cells collected after PBS washing and medium replacement, and TNF-α-stimulated CM pre-incubated with a neutralizing anti-OPG antibody.

**Figure 5:**
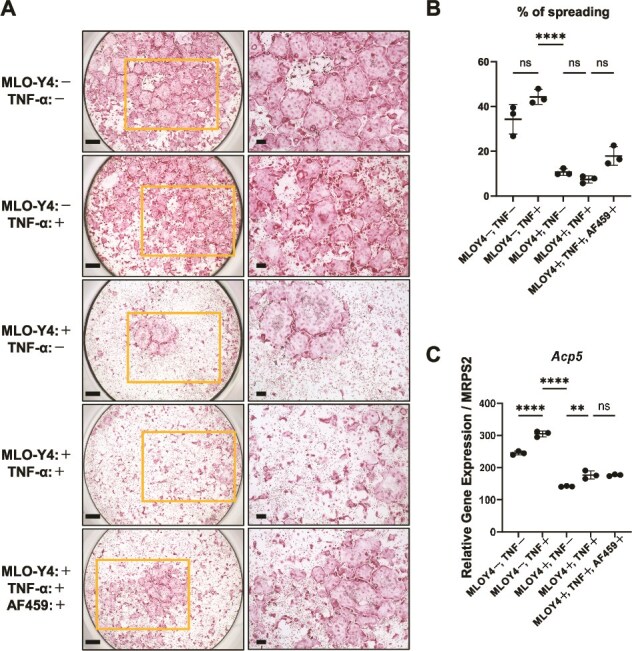
TNF-α-stimulated osteocyte-conditioned medium (CM) suppresses osteoclast differentiation partially via OPG. (A) Representative TRAP staining of osteoclasts generated under 5 conditions: (1) RANKL alone without any TNF-α source or osteocyte-CM (MLO-Y4−, TNF-α−); (2) direct TNF-α treatment in the absence of MLO-Y4 conditioned medium (MLO-Y4−, TNF-α+); (3) conditioned medium (CM) from unstimulated MLO-Y4 cells (MLO-Y4+, TNF-α−); (4) CM from TNF-α-stimulated MLO-Y4 cells collected after PBS wash and medium replacement (MLO-Y4+, TNF-α+); and (5) TNF-α-stimulated MLO-Y4 CM pre-incubated with a neutralizing anti-OPG antibody (AF459). Scale bars are 500 μm for low magnification and 200 μm for high magnification. (B) Dot plots of the percentage of spreading TRAP-positive osteoclasts for the conditions shown in (A) (*n* = 3). (C) Quantitative RT-PCR analysis of Acp5 mRNA expression in osteoclast cultures under the conditions shown in (A). *Acp5* expression levels were normalized to the reference gene (*n* = 3). Data in (B and C) are individual values with mean ± SD; ^**^p < .01, ^****^p < .0001; ns indicates not significant by the one-way ANOVA with Tukey’s post-hoc test.

Representative TRAP staining demonstrated that direct TNF-α treatment resulted in a comparable formation of multinucleated osteoclasts relative to the RANKL-only condition. In contrast, both unstimulated and TNF-α-stimulated osteocyte-conditioned media were associated with a reduced appearance of spread TRAP-positive multinucleated cells, with a tendency toward more pronounced suppression observed in cultures treated with TNF-α-stimulated CM. Pre-incubation of TNF-α-stimulated CM with an anti-OPG neutralizing antibody appeared to attenuate this suppressive morphology (Figure 5A). Consistent with these morphological observations, quantitative analysis of the proportion of the well area occupied by spreading TRAP-positive osteoclasts revealed a significant reduction in CM-treated groups. Partial recovery was observed following anti-OPG antibody treatment; however, this change did not reach statistical significance (Figure 5B). In addition, quantitative RT-PCR analysis showed that expression of the osteoclast lineage marker *Acp5* was significantly increased by direct TNF-α stimulation and significantly decreased by the addition of osteocyte-CM. In contrast, neutralization of OPG in the CM did not result in a statistically significant reversal of this effect (Figure 5C).

Collectively, these results indicate that osteocyte-CM modulates osteoclast morphological maturation and *Acp5* expression under constant RANKL stimulation. However, the present data do not provide evidence that these effects are mediated by a dominant, OPG-dependent mechanism.

## Discussion

This study is an exploratory analysis of osteocyte-associated regulatory factors in surgically obtained femoral head specimens from patients with RA and OA, with the human findings contextualized using an in vitro osteocyte experimental system. Osteoprotegerin protein abundance was increased in osteocyte-enriched femoral neck cortical bone from patients with RA compared with OA, and mechanistic analyses suggest that inflammatory stimulation can enhance osteocytic OPG expression and potentially modulate the osteoclast-regulatory milieu.

Clinically, RA is characterized by systemic bone loss as well as periarticular osteopenia.^2^ In the present study, however, trabecular microarchitectural parameters in the femoral head/neck region did not differ significantly between the RA and OA groups. This apparent discrepancy may be explained by several factors, including the fact that the analyzed region represents a load-bearing site; that, particularly in advanced OA accompanied by pain and functional impairment, reduced mechanical loading can lead to bone loss in the femoral neck^25^ and that OA bone does not represent a “fully healthy” control but is itself subject to disease-associated bone remodeling changes, including alterations in local loading conditions and low-grade inflammatory responses.^26^ Indeed, previous studies comparing bone microarchitecture between RA and OA have reported heterogeneous results, ranging from no significant differences to modest increases.^27^^,^^28^ Accordingly, the human specimen data in the present study should be interpreted as demonstrating differences in OPG abundance within osteocyte-enriched cortical bone between these 2 clinical populations in the specific context of surgical femoral-head specimens, rather than as direct evidence that RA uniformly preserves or worsens trabecular microarchitecture in the hip.

Beyond these structural findings, the present study is notable in that it provides a rare, exploratory analysis of bone metabolism-related proteins in the cortical bone of the human femoral head. In a previous investigation, Lee et al.^29^ analyzed femoral heads from OA and traumatic cases, but did not clearly distinguish cortical from trabecular compartments, leaving open the possibility that proteins derived from multiple cell types were evaluated together. In contrast, to obtain samples as highly enriched for osteocytes as possible, we carefully removed the periosteum and trabecular bone and analyzed the resulting osteocyte-enriched cortical bone. Using this cortical bone protein analysis (including exploratory approaches), we found that OPG levels were significantly higher in the RA group than in the OA group, whereas sclerostin levels were comparable between the 2 groups. Closer inspection at the individual specimen level revealed that some samples exhibited concomitantly low sclerostin and high OPG levels, consistent with patterns previously associated with relative involvement of the Wnt/β-catenin pathway,^30^ whereas other specimens showed high expression of both sclerostin and OPG. The latter pattern suggests the presence of mechanisms capable of inducing OPG independently of the canonical Wnt/β-catenin axis, potentially involving inflammation-related signaling pathways. However, because no significant group differences were observed in trabecular microarchitecture, it is difficult to directly attribute the increase in cortical OPG to disease-specific skeletal phenotypes. Rather, these findings raise the possibility that, in RA, osteocytes mount a counter-regulatory response to inflammatory stimuli by upregulating OPG.^31^

The inflammatory milieu of RA comprises a wide array of cytokines; notably, bDMARD targets TNF-α, IL-6, and IL-17.^2^ The effects of these mediators on osteocytic OPG expression remain controversial. Pathak et al.^13^ reported that ex vivo stimulation of human trabecular bone with individual pro-inflammatory cytokines, such as TNF-α or IL-1β alone, produced little to no change in RANKL or OPG levels, whereas Cawley et al.,^10^ using genetically modified mice, concluded that trabecular protection depends primarily on osteoblast-derived rather than osteocyte-derived OPG. Taken together, these findings imply that cytokine effects vary according to both the bone compartment (cortical vs trabecular) and cell lineage. To address this, we focused specifically on cortical bone osteocytes. Mouse osteocytic cells and OEBFs were exposed to TNF-α, IL-6, or IL-17. Among the cytokines tested, TNF-α induced the most pronounced changes in RANKL and OPG mRNA, and ELISA confirmed a significant increase in OPG protein levels. In RA pathophysiology, diverse cytokines and cellular populations interact in complex ways; accordingly, previous studies have reported OPG changes in the synovium and plasma ranging from reductions to no appreciable change.^32–34^ Some studies have demonstrated that TNF-α stimulation enhances OPG expression in the synovium,^35^ and our data extend this observation to osteocytes.

At the same time, the clinical phenotype of RA—periarticular erosions and systemic bone loss—is largely driven by inflammation-dependent osteoclast activation, with substantial contributions from synovial cell populations. In particular, fibroblast-like synoviocytes can up-regulate membrane-bound RANKL in response to inflammatory cues, providing a potent pro-osteoclastogenic signal at the pannus–bone interface.^36^ In this context, our osteocyte data are compatible with clinical bone loss because TNF-α increased osteocytic RANKL transcripts alongside OPG, and in our system, the induction of RANKL was greater than that of OPG, suggesting a shift toward an increased RANKL/OPG ratio under inflammatory stimulation. Thus, although cortical osteocytes may mount a defensive response by up-regulating OPG, this counter-regulatory mechanism may be insufficient when accompanied by a dominant RANKL increase within osteocytes and/or strong RANKL input from synovial tissues. Collectively, these findings support a model in which osteocyte-derived OPG may attenuate, but not fully prevent, inflammation-driven bone resorption in RA.

Physiologically, osteocytic OPG expression is largely regulated by the sclerostin-Wnt/β-catenin signaling axis.^30^ In the present study, however, our analyses of human femoral neck cortical bone revealed that OPG expression remained high even in some specimens exhibiting elevated sclerostin levels, suggesting that osteocytic OPG regulation cannot be fully explained by the sclerostin/Wnt pathway alone. Moreover, time-course analyses in OEBFs demonstrated that Sost and Wnt target gene expression changed in a non-parallel, time-dependent manner under TNF-α stimulation, underscoring the context-dependent nature of Wnt-related signaling in this ex vivo system. The p38-MAPK pathway is a canonical inflammation-responsive cascade^37^ and has been shown to regulate OPG expression via CREB in BMSCs.^24^ Based on these observations, we hypothesized that a similar mechanism may operate in terminally differentiated osteocytes under inflammatory conditions. Consistent with this hypothesis, TNF-α stimulation induced robust phosphorylation of p38 and CREB in osteocyte-like MLO-Y4 cells, supporting activation of the p38-MAPK-CREB signaling axis. Although TNF-α can activate multiple MAPK pathways, including ERK,^14^^,^^37^ we focused on the p38-MAPK-CREB pathway because of its clear association with CREB activation and downstream transcriptional regulation of OPG. In further support of this notion, pharmacological inhibition of p38 or CREB under TNF-α stimulation was associated with reduced OPG protein levels in MLO-Y4 cells, consistent with a role for p38-MAPK-CREB signaling in the regulation of osteocyte-derived OPG under inflammatory conditions. Furthermore, ChIP analysis demonstrated increased CREB binding to the OPG promoter following TNF-α stimulation, suggesting a potential association between inflammatory signaling and osteocytic OPG transcription. However, given previous reports indicating that osteocyte-specific deletion of OPG has limited effects on cortical bone under physiological conditions,^10^ the functional relevance of inflammation-induced osteocytic OPG remains to be fully elucidated.

In this context, we examined the effects of osteocyte-CM, which contains osteocyte-derived OPG, on osteoclast cultures under constant RANKL stimulation. While CM from both unstimulated and TNF-α-stimulated MLO-Y4 cells modulated osteoclast morphology and gene expression under inflammatory conditions, these effects were not dominantly or exclusively mediated by osteocyte-derived OPG. Neutralization of OPG resulted in only partial attenuation of the observed changes, and the effects did not consistently reach statistical significance. These findings are consistent with previous reports showing limited skeletal phenotypes following osteocyte-specific deletion of OPG under physiological conditions,^10^ while suggesting that osteocytic OPG may acquire functional relevance under inflammatory or stress-related contexts. Rather, the regulation of osteoclast behavior observed in this study is likely to reflect a balance between osteocyte-derived OPG and RANKL, the latter of which may be upregulated in osteocytes in response to inflammatory stimuli, together shaping the osteoclastogenic microenvironment. It should be noted that, although TNF-α increased RANKL mRNA expression in osteocytes, soluble RANKL was not detectable in the CM, consistent with previous reports indicating that RANKL is predominantly expressed in a membrane-bound form.^35^ Taken together, these findings suggest that, under inflammatory conditions, osteocytes can upregulate OPG via p38-MAPK-CREB signaling, potentially serving as a compensatory mechanism to counterbalance inflammation-induced bone resorption.

This study has several limitations. First, the number of human specimens was modest, limiting power for medication-stratified analyses; thus, we cannot exclude contributions of RA therapies and anti-osteoporotic drugs to cortical OPG levels and sclerostin profiles. Second, OA is an imperfect comparator and can exhibit active remodeling and low-grade inflammation.^25^ Therefore, RA-OA differences should not be interpreted as definitive evidence of RA-specific inflammatory regulation. Third, our murine experiments used young adult mice of both sexes without sex-based stratification and acute cytokine stimulation, which do not replicate the chronic, age-matched systemic inflammatory milieu of human RA. Finally, osteocyte-specific OPG genetic models and RA-like in vivo models (eg, TNF transgenic arthritis) were not evaluated and will be required to quantify the contribution of osteocyte-derived OPG to inflammatory bone destruction. Nevertheless, despite the limited sample size, a clear strength of this study is that we performed protein expression analyses on human bone specimens, an area with very few prior reports. Notably, by assessing osteocyte-enriched cortical bone rather than the more heterogeneous trabecular compartment, we provide insights that advance the understanding of osteocyte behavior in the human bone.

In summary, OPG expression was upregulated in the cortical bone of patients with RA, and sclerostin exhibited 2 distinct expression profiles. Among the RA-associated cytokines tested, TNF-α elicited the most robust osteocytic responses and increased OPG at both the transcript and protein levels. Mechanistically, our data support a causal contribution of the p38-MAPK-CREB axis to TNF-α-induced OPG upregulation, as evidenced by pathway activation, enhanced CREB recruitment to the OPG promoter, and pharmacological inhibition experiments. In parallel, TNF-α was accompanied by changes in Wnt/β-catenin-related transcripts, suggesting that homeostatic Wnt signaling may also be engaged in osteocytes under inflammatory conditions, although its direct role in OPG regulation remains to be established. Functionally, factors secreted from TNF-α-stimulated osteocytes—predominantly OPG—suppressed osteoclastogenesis, consistent with the notion that osteocytes can mount a compensatory response that may counterbalance inflammation-driven bone resorption. Collectively, these findings identify the p38-MAPK-CREB pathway as a tractable regulatory axis for osteocytic OPG in an inflammatory milieu and provide a framework for future studies to clarify how inflammatory and homeostatic pathways intersect in cortical bone.

## Supplementary Material

Supplementary_material_ziag023

## Data Availability

The data underlying this article are available in the article.
