# Supplementary material for: Osteocytes regulate osteoprotegerin expression via the p38-MAPK-CREB pathway in rheumatoid arthritis
Source: JBMR Plus. 2026 Feb 17;10(4):ziag023. doi: 10.1093/jbmrpl/ziag023 (PMC12971003; doi:10.1093/jbmrpl/ziag023)
Supplement: Supplementary_material_ziag023 [file supplementary_material_ziag023.docx]

**Supplementary Table1. Comparison of RA patients with vs without b/tsDMARDs**

| Variable | b/ts DMARDs - | b/ts DMARDs + | *P* |
| --- | --- | --- | --- |
| N | 13 | 12 | - |
| Age (years), median (range) | 70 (47-79) | 67 (59-84) | 0.46 |
| Female, N (%) | 10 (76.9) | 12 (100) | 0.08 |
| BMI, median (range) | 24.1 (19.6-30.1) | 23.4 (17.1-30.1) | 0.74 |
| Disease duration (years), median (range) | 4 (1-23) | 23.5 (9-46) | <0.01 |
| RF (IU/mL), median (range) | 17 (5-220) | 64 (7-116) | 0.67 |
| CRP (mg/L), median (range) | 0.31 (0.04-8.26) | 0.08 (0.01-0.61) | 0.02 |
| Steinbrocker stage, N (%) | IV: 1 (10)  III: 2 (20)  II: 5 (50)  I: 2 (20) | IV: 3 (25)  III: 4 (33)  II: 5 (42)  I: 0 (0) | 0.32 |
| Treatment |  |  |  |
| MTX use, N (%)  MTX dose (mg), median (range) | 6 (46)  0 (0-10) | 8 (67)  4 (0-16) | 0.30  0.49 |
| GC use, N (%)  GC dose (mg), median (range) | 5 (38)  5 (1-5.0) | 4 (33)  6 (3-13) | 0.79  0.29 |
| Anti-Osteoporosis drug use, N (%) | 3 (23) | 4 (33) | 0.57 |
| Bone microarchitectural parameters |  |  |  |
| BV/TV (%), median (range) | 37.8 (14.2-64.8) | 33.6 (26.3-56.0) | 0.91 |
| Tb. Th (µm), median (range) | 342 (286-448) | 342 (244-459) | 0.97 |
| Tb. N (/µm), median (range) | 0.62 (0.30-1.01) | 0.62 (0.51-1.00) | 0.76 |
| Tb. Sp (/µm), median (range) | 420 (301-737) | 421 (306-511) | 0.44 |
| Relative Expression of Proteins |  |  |  |
| OPG/beta-Actin, median (range) | 1.49 (0.14-7.55) | 1.69 (1.00-4.5) | 0.55 |
| Sclerostin/beta-Actin, median (range) | 0.32 (0.01-7.71) | 0.84 (0.11-8.02) | 0.28 |

b/ts DMARDs, bio/targeted synthetic Disease-Modifying Anti-Rheumatic Drugs; BMI, body mass index; RF, rheumatoid factor; CRP, C-reactive protein; MTX, methotrexate; GC, glucocorticoid; BV/TV, Bone volume per Tissue volume; Tb. Th, Trabecular bone Thickness; Tb. N, Trabecular bone Number; Tb. Sp, Trabecular bone Space; OPG, Osteoprotegerin.

**Supplementary Table2. Comparison of RA patients with vs without antiresorptive drugs**

| Variable | antiresorptive drug- | antiresorptive drug+ | *P* |
| --- | --- | --- | --- |
| N | 18 | 7 | - |
| Age (years), median (range) | 68 (47-84) | 75 (57-79) | 0.47 |
| Female, N (%) | 15 (83.3) | 7 (100) | 0.25 |
| BMI, median (range) | 24.5 (17.1-30.1) | 23.4 (19.7-29.2) | 0.56 |
| Disease duration (years), median (range) | 11 (1-46) | 10 (2-32) | 0.92 |
| RF (IU/mL), median (range) | 20 (7-220) | 64 (5-100) | 0.90 |
| CRP (mg/L), median (range) | 0.18 (0.01-8.26) | 0.10 (0.02-0.61) | 0.47 |
| Steinbrocker stage, N (%) | IV: 4 (27)  III: 2 (13)  II: 7 (47)  I: 2 (13) | IV: 0 (0)  III: 4 (57)  II: 3 (43)  I: 0 (0) | 0.10 |
| Bone microarchitectural parameters |  |  |  |
| BV/TV (%), median (range) | 33.7 (14.2-64.8) | 33.6 (28.6-56.5) | 0.65 |
| Tb. Th (µm), median (range) | 333 (274-456) | 351 (244-459) | 0.88 |
| Tb. N (/µm), median (range) | 0.63 (0.30-1.02) | 0.62 (0.55-1.00) | 0.46 |
| Tb. Sp (/µm), median (range) | 423 (301-737) | 400 (306-524) | 0.37 |
| Relative Expression of Proteins |  |  |  |
| OPG/beta-Actin, median (range) | 1.69 (0.14-7.55) | 1.54 (0.61-1.94) | 0.58 |
| Sclerostin/beta-Actin, median (range) | 0.57 (0.01-8.02) | 1.41 (0.12-7.71) | 0.47 |

“Antiresorptive drugs” are defined as bisphosphonates, SERMs, and denosumab; rPTH, vitamin D, and romosozumab are excluded.

b/ts DMARDs, bio/targeted synthetic Disease-Modifying Anti-Rheumatic Drugs; BMI, body mass index; RF, rheumatoid factor; CRP, C-reactive protein; MTX, methotrexate; GC, glucocorticoid; BV/TV, Bone volume per Tissue volume; Tb. Th, Trabecular bone Thickness; Tb. N, Trabecular bone Number; Tb. Sp, Trabecular bone Space; OPG, Osteoprotegerin.

**
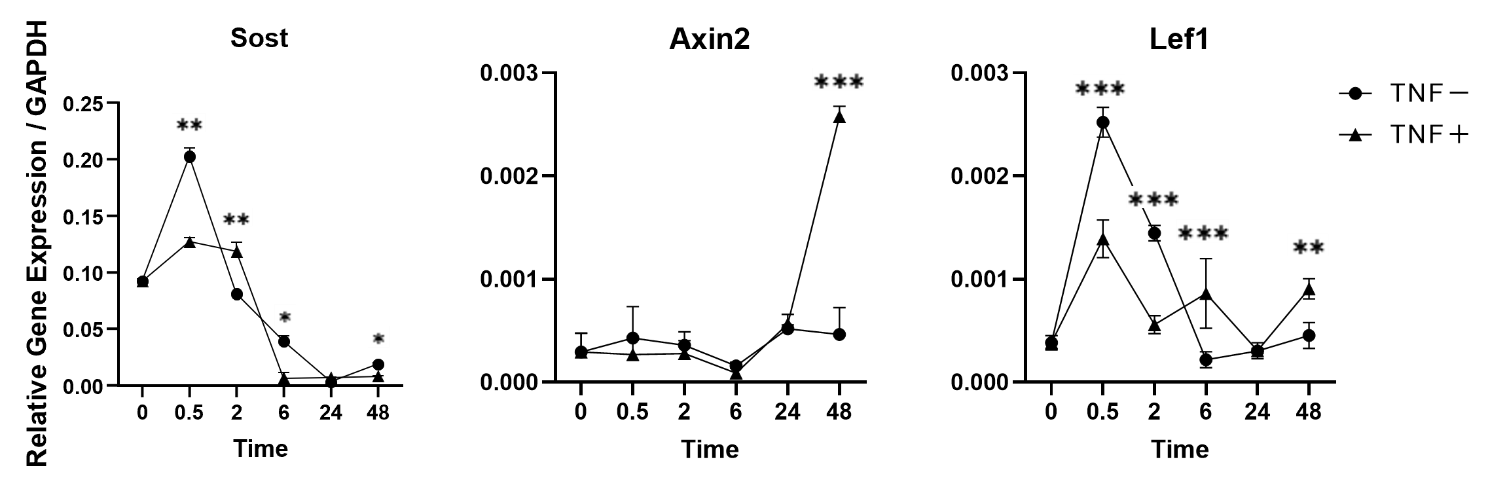
**

**Supplementary Figure 1.** Time-course analysis of *Sost*, *Lef1*, and *Axin2* mRNA expression in osteocyte-enriched bone fractions (OEBFs) stimulated with or without TNF-α (n = 3).

Data are presented as mean ± SD and were analyzed by two-way ANOVA with time and TNF-α treatment as independent factors, followed by Šídák’s multiple comparisons test to compare TNF-α–treated and untreated groups at each time point.

*P < 0.05, **P < 0.01, ***P < 0.001.
